# Study of Toughness and Macro/Micro-Crack Development of Fibre-Reinforced Ultra-High Performance Concrete After Exposure to Elevated Temperature

**DOI:** 10.3390/ma12081210

**Published:** 2019-04-12

**Authors:** Piotr Smarzewski

**Affiliations:** Department of Structural Engineering, Faculty of Civil Engineering and Architecture, Lublin University of Technology, 20-618 Lublin, Poland; p.smarzewski@pollub.pl; Tel.: +48-81-538-43-94

**Keywords:** ultra-high performance concrete, crack development, flexural toughness, elevated temperature, steel fibre, polypropylene fibre

## Abstract

This study has investigated the changes that might appear in post-peak flexural response. Before the flexural test, prismatic specimens were placed in a furnace chamber and exposed to elevated temperatures of 400, 600, and 800 °C. The flexural toughness test was carried out on two types of concrete: Plain ultra-high performance concrete (UHPC) and UHPC with different types of fibres (steel fibre (SF) and polypropylene fibre (PPF)) at 0.5%, 1%, 1.5%, and 2% volume fractions. During the flexural test in the macro-crack development analysis, the non-contact ARAMIS system was used to perform three-dimensional measurements of strain and displacement. The results of scanning electron microscope (SEM) observations of micro-crack development in UHPC without and with SF/PPF were also presented. The experimental results showed that in some cases, the load–deflection curve of fibre-reinforced UHPC displayed a double-peak response. The first peak signified the UHPC properties, while the second peak represented the properties of the fibres. Under flexural load, the toughness decreased as the temperature increased. Significant decrease in the load–deflection response and toughness were observed for the polypropylene fibre-reinforced UHPC when the temperature approached 800 °C. The SEM observation results showed that the thermal damage of fibre-reinforced UHPC depends on the pore pressure effect, the thermal mismatch, the decomposition of hydration products, and the formation of micro-cracks.

## 1. Introduction

Concrete for special applications such as high-rise buildings, nuclear power plants, tunnels, radioactive waste storage facilities, and military and underground structures should be characterised by high temperature resistance, energy absorption capability, and durability [1,2]. Explosive spalling of concrete at high temperatures is a complex problem, as a result of which, after concrete cracking, the concrete cover is spalled and the steel reinforcement is exposed to fire, which is dangerous due to the load-carrying capacity of the structure [3,4]. 

Ultra-high performance concrete (UHPC) has high compressive strength, which is at least 120 N/mm^2^ [5,6], or according to other researchers 150 N/mm^2^ [7,8,9], as well as increased durability and high ductility [10]. This material is used to improve the bearing capacity of pedestrian footbridges, highway bridges, and the precast lattice-style facade system for buildings and stadiums [10,11]. Its sensitivity to explosive spalling in fire conditions results mainly from its dense structure and low permeability in comparison with normal-strength concrete [12]. These properties make it difficult to release vapour pressure, which leads to physical damage [13]. This problem is particularly important at high heating rates [14]. Introducing polypropylene fibres (PPF) to concrete mixtures is a widely accepted and used method to control and limit explosive spalling [15,16,17]. It was found that the aggregate size also has a significant impact on the concrete spalling intensity, since the usage of aggregates with a larger grain size implies the formation of porous interface transition zones (ITZ) [18]. Therefore, it has been proposed to link these zones by PPF which, after melting at elevated temperatures, will form tunnels through which water vapour will be released [19,20,21]. This solution can be effective in UHPC, which is a very dense material due to the use of aggregates of a fine fraction and silica fume at a low water-to-binder ratio [22]. Heinz and Ludwig found that UHPC reinforced with PPF at a content of 0.6 vol% has a weight 9% lower after exposure to a temperature of 1000 °C [21]. In other studies, Liu and Huang [23], and Tai et al. [24] noticed an increase in the compressive strength of UHPC samples heated up to 300 °C. Conversely, a decrease was reported at higher temperatures. After testing the fire duration at a constant temperature of 500 ± 50 °C, the UHPC compressive strength decreases—after 60 min of fire it decreases to 62%, and after 120 min it is 56% of the original compressive strength [23]. A drop in compressive strength at about 400 °C is given in another reference [25]. In turn, the axial tensile strength of UHPC with steel fibres (SF) decreases at 200 °C, increases in the range of 200–300 °C, and falls above 300 °C. Below 600 °C, the axial tensile strength of UHPC increases, and then it decreases between 600 and 800 °C as the steel fibre content increases [26]. It was also observed that after exposure to high temperatures, cracks of 0.3—0.5 mm in width were formed on the surfaces of the samples [13]. It was also found that the mechanical properties of UHPC decrease due to its chemical decomposition, dehydration of calcium silicate hydrate products, and expansive thermal damage [27,28]. Other studies have reported that the addition of steel fibres might reduce explosive spalling and raise the fire resistance of high-strength concrete (HSC) columns [15]. On the other hand, it was noted that in the very dense UHPC microstructure, their use is not that effective anymore [29]. Additionally, mixtures of various steel fibres, as well as polypropylene and steel fibres, were successfully applied to inhibit the spalling of self-compacting concrete, HSC, and UHPC [12,14,17,30,31]. Despite the fact that the UHPC explosive spalling mechanism is not fully recognised, two main hypotheses are presented in the literature. One hypothesis is that the decisive factor in spalling is the build-up of pressure in the pores, which generates tensile stresses exceeding the tensile strength of the matrix [14,30,32,33]. On the other hand, another hypothesis assumes that the increase in strain energy is caused by the thermal incompatibility between the aggregate grains and the cement mortar [14,32,34].

The effects of high temperature on the physical and mechanical properties of steel/polypropylene fibre-reinforced UHPC were investigated by some researchers [13,24,25,26,27,28,30,31]. However, the compressive strength or tensile splitting strength measured at the first peak may be insufficient to describe the behaviour of the UHPC with fibres, as the conclusions based on testing the peak strength values may be misleading. In contrast, the flexural toughness test is an appropriate choice to obtain information about the properties of the fibres because the main purpose of their application in UHPC is to improve the material’s ability to absorb energy and transfer loads after cracking. For this reason, the flexural toughness and fracture energy will provide information and enable the properties and roles of different types of fibres in UHPC after high temperature exposure to be determined. An exhaustive literature review indicated that no studies in the literature on the flexural toughness and fracture energy of UHPC with SF/PPF after being subjected to high temperatures are currently available. 

The present study has investigated the flexural toughness and fracture energy of UHPC with various fibre contents after withstanding high temperatures. Before the flexural test, prismatic samples were placed in a furnace chamber and exposed to elevated temperatures of 400, 600, and 800 °C using ISO/TR834 standards [35]. The flexural toughness test was carried out on two types of concrete: Plain UHPC and UHPC with single fibre types (SF or PPF) at 0.5%, 1%, 1.5%, and 2% volume fractions. The differences in toughness indexes and fracture energies of UHPC with the two types of fibres at several fibre contents after being subjected to high temperatures are compared. In the macro-crack development analysis performed during the flexural test, the non-contact ARAMIS system for three-dimensional measurements of strain and displacement was used [36]. Finally, scanning electron microscope (SEM) observation results of the micro-crack development in plain, and SF- and PPF-reinforced UHPC were also presented and discussed. The results of this study provide a valuable reference for future structure applications and design. 

## 2. Experimental Procedure

### 2.1. Materials and Specimens Preparation

Nine UHPC mixtures were prepared using Portland cement type I—CEM I 52.5 N-HSR/NA (CEMEX, Chełm, Poland), condensed silica fume, quartz sand and basalt aggregates with maximum sizes of 2 and 16 mm, respectively, tap water, steel fibres BAUMIX® (BAUTECH®, Piaseczno, Poland) or polypropylene fibres BAUCON® (BAUTECH®, Piaseczno, Poland), and a superplasticiser CX ISOFLEX 793 (CEMEX Admixtures GmbH, Salzkotten, Germany). These components had been found appropriate to produce fibre-reinforced UHPC in previous investigations [7,37,38]. The physical properties and chemical composition of the cement and condensed silica fume are given in Table 1. 

The specific gravity and water absorption of the basalt coarse aggregate were 2.95 and 0.15%, respectively, and those of the quartz sand were 2.67 and 1.12%, respectively. The sieve analyses of the aggregates are given in Table 2. 

**Figure 1:**
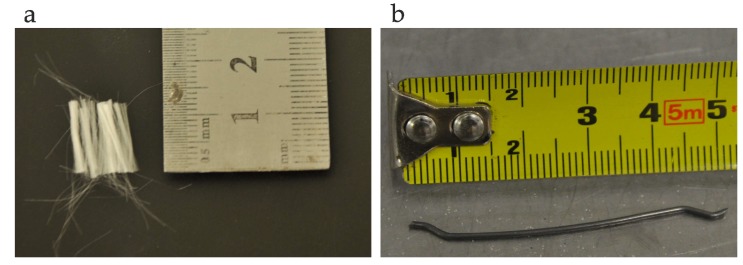
(**a**) Polypropylene fibres and (**b**) steel fibres.

Two types of fibre were used: Steel and polypropylene at four different volume fractions of 0.5%, 1%, 1.5%, and 2%. The hooked-end steel fibre characteristics were: Length/diameter = 50 mm/1 mm, specific gravity = 7.8, and tensile strength = 1000 N/mm^2^. The crimped polypropylene fibre properties had the following parameters: Length/diameter = 12 mm/25 µm, specific gravity = 0.9, and tensile strength = 350 N/mm^2^. The fibres are shown in Figure 1. 

A polycarboxylate ether-based light yellow liquid superplasticiser, expressed as 3% of the mass of cement, was employed in the UHPC mixtures. The superplasticiser had a specific gravity of 1.07 at 20 °C, contained chlorides < 0.1%, alkali < 1.5%, and did not induce air entrainment. 

The reference slump for the control mixture was 120 ± 20 mm. The slump test and unit weight test for workability were performed on fresh UHPC. The mixing parameters for the UHPC are presented in Table 3. 

Mixing of the UHPC was done by dry mixing the sand and basalt for 3 min, after which 50% of the water was added to the dry mix. The wet blend was mixed for a further 2 min before the remaining water, superplasticiser, cement, and silica fume were added to the mixture and mixed thoroughly for about 3 min. The fibres were then manually added to the mixture, with care being taken to ensure there was no balling of the fibres. The UHPC was ready for casting after 4 min of mixing. The concrete was placed in moulds and compacted using a proctor vibrator. The specimens were left undisturbed in the moulds for 24 ± 5 h before demoulding, and then were placed in water with a regulated temperature of 20 ± 2 °C for 14 days. Then, the specimens were kept at constant room temperature until testing. The number of beam specimens tested was six specimens from the plain concrete (C0) mixture (three in two different temperatures), twelve specimens from each CPP batch-mixture (three for each temperature), and nine specimens from each CS batch-mixture (three for each temperature). All other tests were performed on a set of three samples for each mixture. However, it was assumed that the individual result variation of a set of specimens should not be more than 10% of the average. If a greater variability was achieved, the test result of the specimens was not considered and the test was repeated. The mean value obtained from these specimens was used to evaluate the mixture design.

### 2.2. Test Set-Up

**Figure 2:**
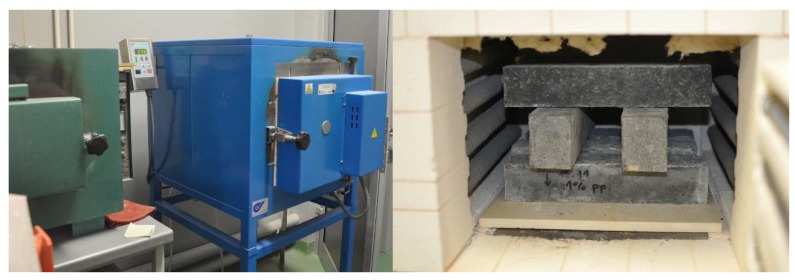
Specimens in oven chamber.

**Figure 3:**
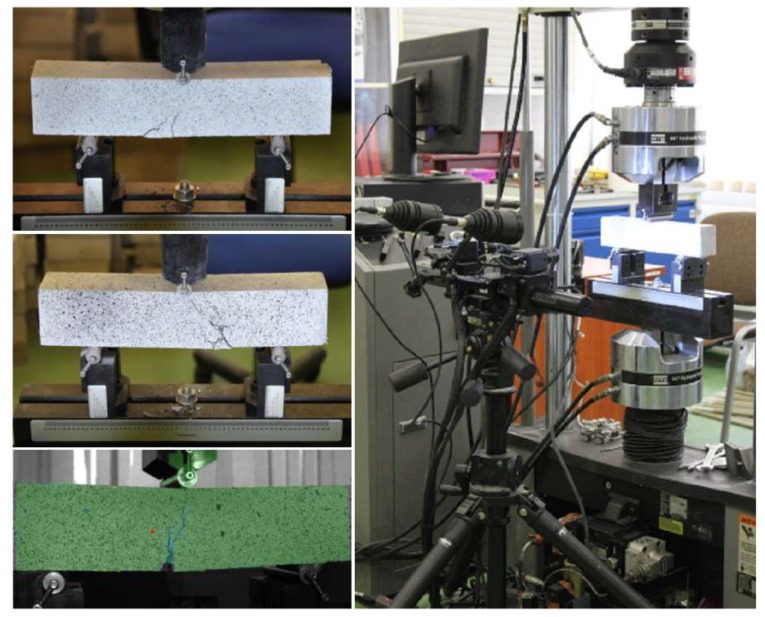
Flexural toughness set-up.

Tests of the apparent density, absorbability, and open porosity were carried out on cubic specimens with the dimensions 100 × 100 × 100 mm according to EN 12390-7:2009 [39] and EN 13755:2008 [40]. Compressive and tensile strength, as well as elastic modulus tests of the UHPC at room temperature were conducted in Walter-Bai AG and Advantest 9 hydraulic presses within 3 × 10^3^ N, at 28 ± 2 days of curing. The compressive and tensile splitting strength tests were realised according to the recommendations in the standards PN-EN 12390-3:2011 [41] and PN-EN 12390-6:2011 [42] on cubes with sides of 100 mm, respectively. The elastic modulus tests were realised on cylinders with a height/diameter of 300/150 mm in the Walter-Bai AG press on the basis of ASTM C469/C469M-14 [43]. The flexural strength test was carried out in accordance with the PN-EN 12390-5:2011 normative [44] on beam specimens with the dimensions 50 × 50 × 250 mm (CPP) and 50 × 63 × 350 mm (CS, C0) under a three-point scheme of loading at the rate of 0.05 N/(mm^2^s). The UHPC flexural strength decreases with increasing specimen size [11]. It was found that the 700 × 150 × 150 mm specimen showed a 33% lower flexural strength compared to a 160 × 40 × 40 mm specimen [45]. It was also observed that smaller specimens tended to create favourable 2D patterns of fibre orientation along the cross-section, which leads to higher flexural strength [46]. The recommended reduction factor is 9%, with a difference in the specimen height of 50 mm [47]. In the present study, it was assumed that the effect of the specimen size on flexural toughness can be neglected. Prior to the flexural tests, the beams were placed in an oven chamber (Figure 2). The temperature was increased according to the ASTM E 119-98 standard temperature-time curve [48]. The CPP beams were exposed to the following temperatures: 400, 600, and 800 °C. On the other hand, the C0 beams were only exposed to the temperature of 400 °C, and the CS beams to the temperatures of 400 and 600 °C, in order to prevent the occurrence of UHPC spalling. After removing from the oven, the beam specimens were left to cool for 24 h and then subjected to a three-point bending test. The experimental work was performed with a servo-hydraulic testing machine MTS 810 (MTS, Eden Prairie, MN, USA) having the static force capacity of 120 kN. The results in terms of load–deflection were recorded and then toughness indexes were calculated according to ASTM C1018 [49]. In the investigation of the flexural toughness of the UHPC, the non-contact ARAMIS system for three-dimensional measurements of the deformation was used (Figure 3), as described in references [50,51,52]. The qualitative analysis of the micro-crack development and pores distribution in the UHPC was determined using a scanning electron microscope (SEM) FEI Quanta 250 FEG (Thermo Fisher Scientific, Hillsboro, OR, USA). The specimens were prepared as thin-layer plates for each UHPC mixture.

## 3. Results and discussions

### 3.1. Properties of UHPC 

When the steel and polypropylene fibres were added to the mixtures, a decrease in the slump values was observed with the increasing amount of fibres (Table 3). Furthermore, it was determined that the unit weight of the fresh UHPC also decreases with the increasing steel and polypropylene fibre content.

The obtained mean results of the apparent density, absorbability, open porosity, compressive strength, splitting tensile strength, flexural strength, and elastic modulus of the UHPC at 28 days are given in Table 4.

An increase in the polypropylene fibre volume content from 0.5% to 2% affects decreases in the density and open porosity of the UHPC up to 11% and 12%, respectively, and an increase in absorbability of about 33%. On the other hand, the results show that the increase in steel fibre content has an influence on gradual increases in the density, absorbability, and open porosity up to 2%, 45%, and 25%, respectively. Adverse effects of the addition of both types of fibres on the UHPC compressive strength at 28 days can be found. The addition of polypropylene and steel fibres results in compressive strength decreases of about 22% and 4%, respectively, compared to the compressive strength of plain UHPC. It can be assumed that the fibre–matrix connection zones are weaker areas in the UHPC, in which poor void regions may occur, through which the failure will initiate. The use of higher amounts of soft polypropylene fibres causes their clustering and the formation of a more porous and fragile UHPC matrix, which results in the highest decrease in compressive strength. In another investigation, it was also observed that polypropylene fibre-reinforced concrete has poorer workability, a larger portion of entrapped air, and a lower compressive strength than concrete without fibres [53]. On the other hand, the lowest decrease in strength was observed when 2% hard steel fibres were added (Table 4). An increase in compressive strength can be possible by adding shorter steel fibres with lengths of 10–15 mm, a higher aspect ratio, and higher strength. The mean tensile splitting and flexural strengths decrease with increasing the addition of polypropylene fibres. However, both strengths increase with a higher content of steel fibres, up to a 1.5% fibre content. The addition of PPF resulted in a decrease in the elastic modulus. An adverse influence of the addition of SF on the UHPC modulus of elasticity is observed.

### 3.2. Flexural Behaviour 

**Figure 4:**
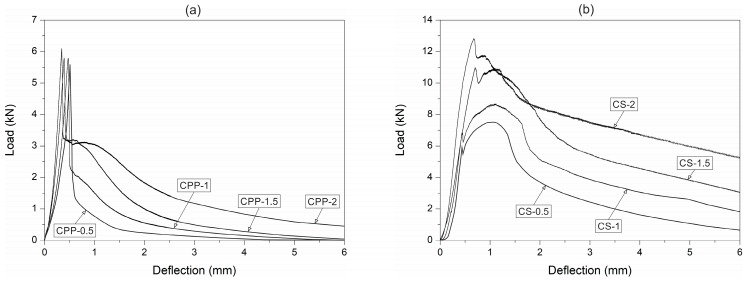
Load–deflection response of: (**a**) CPP and (**b**) CS fibre concretes at room temperature.

The mean peak load of plain UHPC at room temperature was 10.2 kN. The flexural strength at a temperature of 400 °C decreased by 32%. The decrease in flexural strength was probably due to the cracks appearing in the UHPC because of the differing thermal expansion coefficients between the cement paste, coarse aggregates, and fine aggregates. The C0 beam specimens were only exposed to the temperature of 400 °C, in order to avoid UHPC explosive spalling. Explosion of the C0 specimens occurred at nearly 450 °C. The phenomenon is ascribed to dehydration of the calcium hydroxide that appears above 400 °C, which increases the build-up of high pressure in small pores. This is in accordance with another study carried out for high-strength concrete under high temperatures [54]. The typical responses of the CPP (with a fibre content greater than or equal to 1.5%) and CS fibre-reinforced UHPC at room temperature presented a two-peak response (Figure 4). The first peak showed the flexural strength of the UHPC and the first flexural cracking load. On the other hand, the second peak indicated the ability of the fibres to sustain increases in load after the first flexural crack occurred. The peak loads of the CPP and CS concrete at room and high temperatures are given in Table 5.

A significant drop in the flexural strength occurred after the first peak for the polypropylene fibres UHPC specimen, even with the highest fibre content. It was due to the low strength of the polypropylene fibres. In the case of the steel fibre-reinforced UHPC, the double-peak response appeared due to the high strength and stiffness of the steel fibres. Nevertheless, the load drop after the first crack was lower when the steel fibre content was increased. The second peak of the load–deflection curve is characterised by the ability of the fibres to resist load after crack initiation. The steel fibre UHPC gives the impression of performing better than the polypropylene fibre UHPC, as observed by the higher post-peak load. 

**Figure 5:**
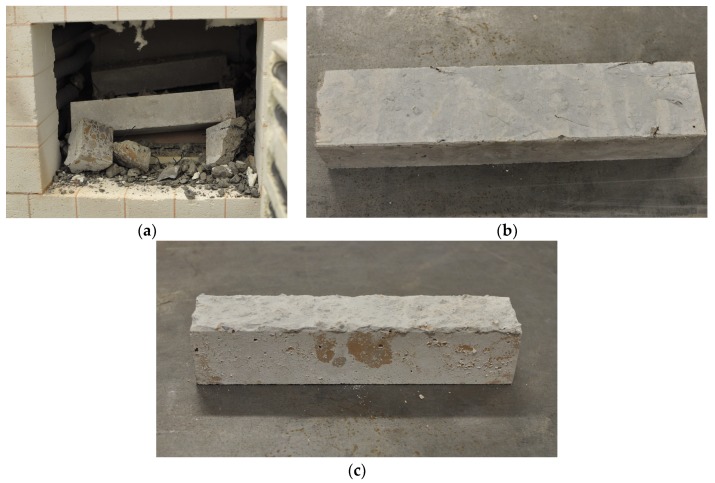
Failure of flexure specimens due to high temperature. (**a**) Spalling of CS-1.5 at 650 °C, (**b**) cracking of CS-0.5 at 900 °C, and (**c**) cracking of CPP at 1000 °C.

Explosive spalling did not occur in the CS and CPP specimens with each tested fibre volume content heated to 600 and 800 °C, respectively. The CS specimens with a fibre content above 1% suffered explosive spalling damage when exposed to nearly 650 °C. The failure of the CS specimens was clearly audible in the laboratory due to the strong explosion. Figure 5a shows an example of the observed CS specimen failure when submitted to the temperature of 650 °C. On the other hand, the CS specimens with 0.5 and 1 vol% fibre contents did not exhibit any spalling at 900 °C (Figure 5b). By contrast, the CPP mixtures behaved differently at elevated temperatures. Explosive spalling did not take place when the temperature at the surface of the CPP specimens was about 1000 °C (Figure 5c). 

**Figure 6:**
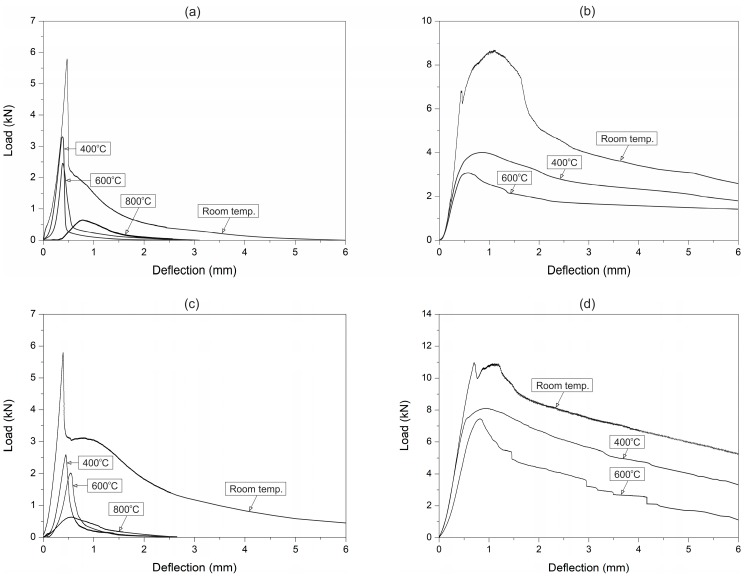
Flexural responses of (**a**) CPP-1, (**b**) CS-1, (**c**) CPP-2, and (**d**) CS-2 concretes at high temperature.

The fibre-reinforced UHPC flexural tensile response after temperature exposure is shown in Figure 6. Both in the CS and CPP concrete at the temperature of 400 °C, two variations were observed. First, the beam specimen stiffness decreased, and secondly, the post-peak tensile response significantly deteriorated. The beam stiffness decrease was due to the reduced concrete stiffness after its exposure to high temperature. Heat can initiate and accelerate hydration between free cement particles and water around the interfacial zone, and finally, the fibre–cement paste bond strength may increase. Both the stiffness and the first peak load started to decline slightly at the temperature of 600 °C. At 800 °C, the high temperature caused the polypropylene fibres to evaporate in the CPP concretes. For the steel fibre-reinforced UHPC, the temperatures were not to high enough to cause the steel fibres to melt, but their colour changed to black. The load response was also different after heating to 600 °C, but at a lower level than that of the polypropylene fibre UHPC, due to the fact that the steel fibres did not evaporate and they were able to provide the required load resistance. The drop in flexural strength was not as noteworthy as that of the polypropylene fibre UHPC. The steel fibre-reinforced UHPC, unlike the PPF-reinforced UHPC, did not lose much of its strength at 600 °C (see Figure 6). Since the steel fibres did not melt or evaporate at this temperature, all of them remained intact and helped in carrying the load. Another thing to notice from the load–deflection response is the disappearance of the first peak. This indicated that the fibres had taken over the loading from the beginning, because the concrete strength was low.

### 3.3. Flexural Toughness and Fracture Energy Evaluation 

**Figure 7:**
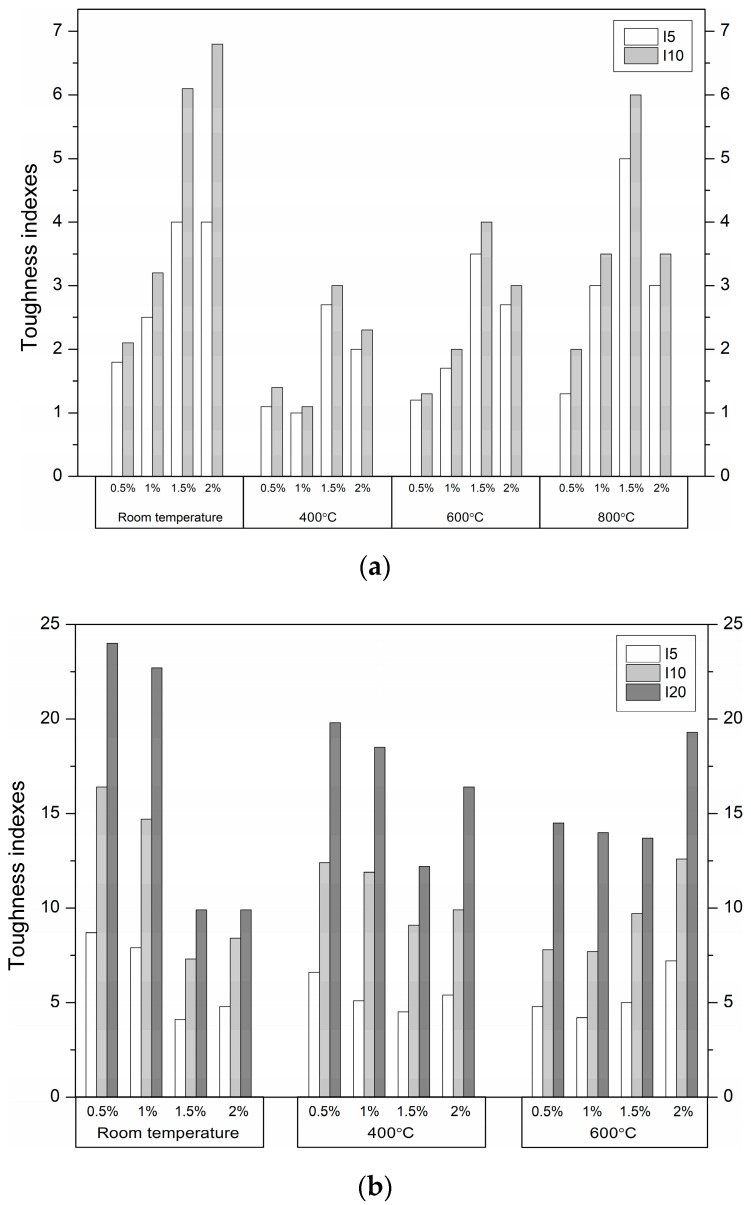
Toughness indexes of (**a**) polypropylene fibre (PPF)- and (**b**) steel fibre (SF)-reinforced UHPC.

Flexural toughness is defined as the post-crack energy absorption ability of fibre-reinforced concrete. The toughness indexes at I5, I10, and I20, defined according to ASTM C1018 [49], were calculated using the areas under the load–deflection curve up to specified deflections, obtained from the toughness tests (Table 5). Deflection up to the first crack *δ*, and 3*δ*, 5.5*δ*, and 10.5*δ* are identified. Generally, fibre-reinforced UHPC produced from UHPC with the same strength will exhibit a similar first crack load. This is because the first crack depends on the plain UHPC tensile strength. The fibre-reinforced UHPC flexural toughness up to the first crack at room temperature was within the range 0.7–4.3 kN × mm. The fibre-reinforced UHPC exhibited lower first crack toughness at high temperatures than at room temperature. The strength of the plain UHPC decreased considerably, and the stiffness dropped evidently as well. The peak response refers to UHPC because the fibres are inactive prior to cracking. The toughness decreases beyond the first peak. In this case, it resulted from the degraded interfacial bond strength between the steel fibres in the CS mixtures or the polypropylene fibres melting in the CPP mixtures. The first peak toughness of the fibre-reinforced UHPC at 600 °C was 2–3.5 and 1.7–3.6 times lower compared to the corresponding values obtained for the CPP and CS specimens tested at room temperature, respectively. In general, the largest decreases were obtained by the specimens with the highest fibre volume content. The toughness dropped due to the decrease in first crack strength and stiffness in all the fibre-reinforced UHPC specimens. The polypropylene fibre-reinforced UHPC lost its load capacity by significantly reducing the toughness. These fibres began to evaporate at 170 °C. When the temperature reached 600 °C, most of the polypropylene fibres had completely melted and left numerous empty channels, and then the UHPC lost its mechanical properties (the evidence is provided in Section 3.5). By contrast, the steel fibres did not melt at this temperature and still helped in carrying the load. However, it can be observed that the first peak disappeared from the load–deflection curve. This indicated that the UHPC matrix strength was very low. At 800 °C, when the polypropylene fibre content increased, the loss of toughness was more evident. The influence of the fibre content on the toughness indexes of the fibre-reinforced concrete at different temperatures is shown in Figure 7.

The toughness index values depend mainly on the type, volume content, and aspect ratio of the fibres, and are essentially independent from the concrete matrix. Therefore, the indexes reflect the toughening effect of the fibres. In general, hooked-ends steel fibres produce toughness indexes greater than those for smooth straight polypropylene fibres at the same volume content and temperature. The index values of 5 for I5 and 10 for I10 indicate a composite with plastic behaviour after the first crack at steel fibre volumes of 1% or less. Lower fibre volume contents or less effectively anchored fibres produce respectively lower index values [55]. For the UHPC containing the with-hooked-end steel fibres, index values of 7.9–8.7 for I5 and 14.7–16.4 for I10 are achieved at fibre volumes of 0.5%–1% at room temperature. When at least a 1.5% steel fibre content was added to the matrix, the flexural strength of the UHPC increased. However, the molecular continuity of the matrix was broken, and the micro-cracks increased when the SF content was high. Thus, the toughness indexes decreased at room temperature with an increased SF content. After the matrix was damaged due to heating, the SF content had a decisive influence on the obtained values of the toughness indexes. On the other hand, for the UHPC containing the straight PPF, toughness index values of 4 for I5 and 6.1–6.8 for I10 are attained at the PPF content of 1.5%–2% at room temperature. The toughness indexes of the UHPC reinforced with PPF increase more slowly than those of UHPC reinforced with SFs, due to the fact that they are less efficiently anchored. Furthermore, a high content of PPFs in UHPC leads to porosity, micro-cracks, and local fibre aggregation. The inadequate cohesion of PPFs with the UHPC leads to insufficient stress transfer. Therefore, the toughness indexes do not increase linearly with increasing PPF content.

**Figure 8:**
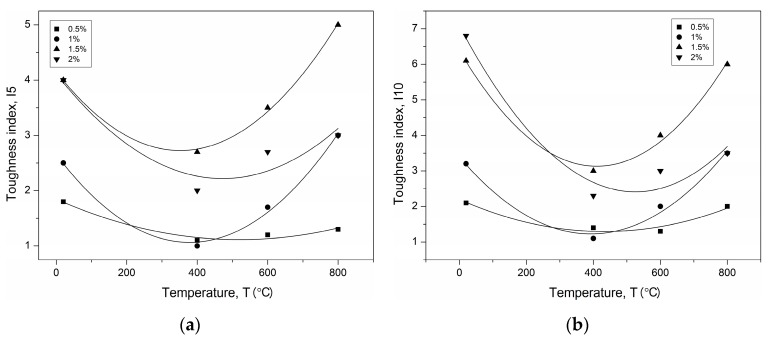
Temperature–toughness indexes fitted curves of PPF-reinforced UHPC (**a**) I5, (**b**) I10.

**Figure 9:**
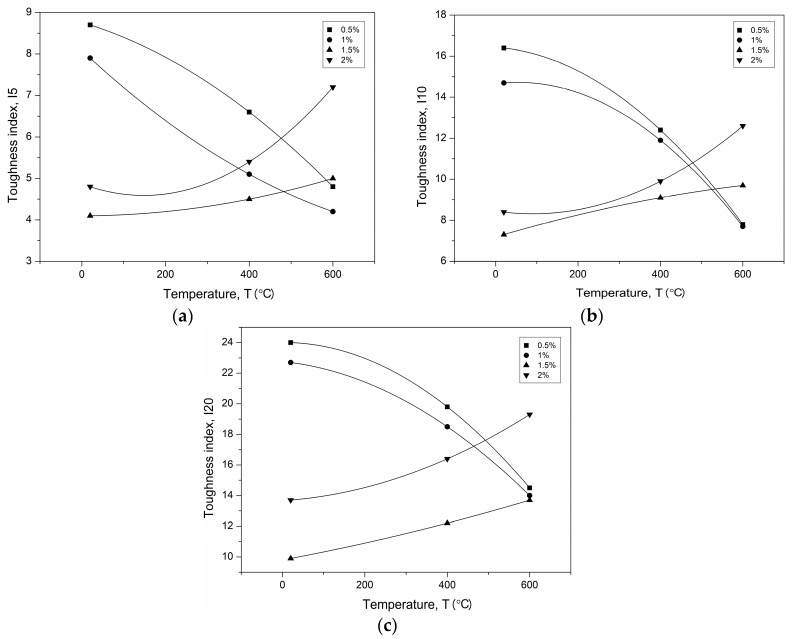
Temperature–toughness indexes fitted curves of SF-reinforced UHPC (**a**) I5, (**b**) I10, (**c**) I20.

The values obtained for the toughness indexes of I5, I10, and I20 for PPF- and SF-reinforced UHPC are shown in Figure 8 and Figure 9 as a function of temperature.

It can be concluded that the nonlinear relations agree very well with the test results. Nonlinear regression leads to the relationships of the temperature–toughness indexes listed in Table 6. 

**Figure 10:**
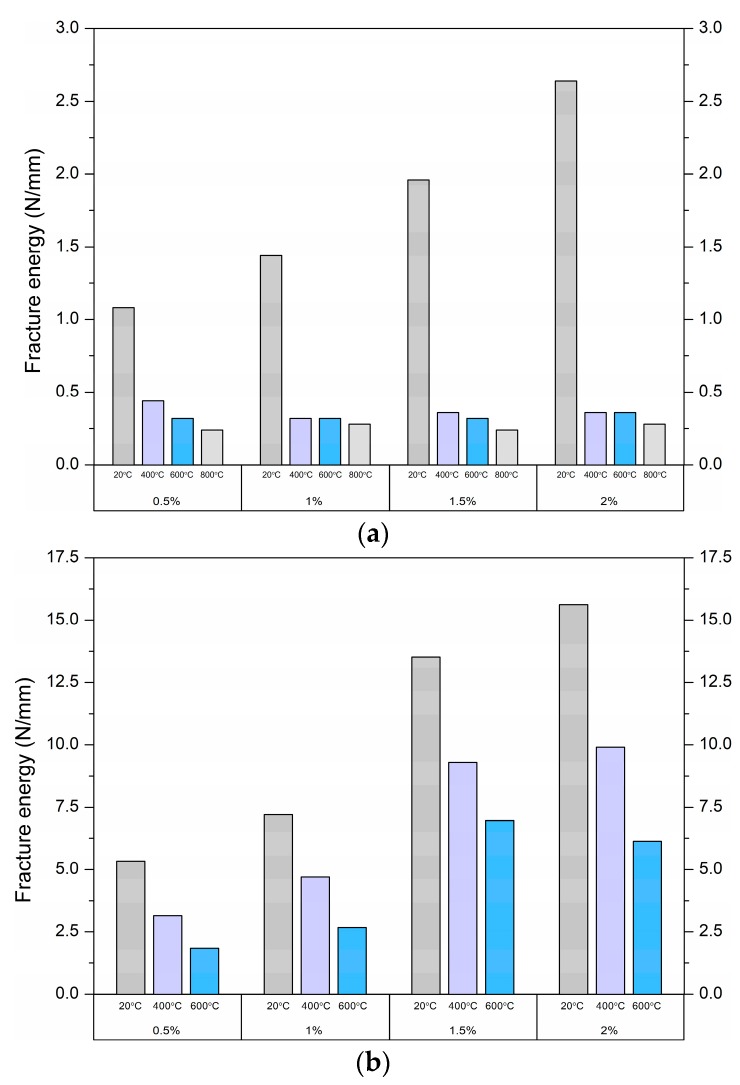
Fracture energy of (**a**) PPF- and (**b**) SF-reinforced UHPC.

Fracture energy is computed by integrating the total area under the load–deflection curve of the specimen without a notch, divided by the total crack ligament area (equal to the cross-section of the specimen) [56]. The fracture energy values of each test series are shown in Table 5 and Figure 10.

Hooked-end steel fibres produce higher fracture energy than straight polypropylene fibres. For SF-reinforced UHPC, fracture energies of 5.33–15.62 are achieved at fibre volumes of 0.5%–2% at room temperature. After the matrix failures due to heating, the content of SF has a major influence on the fracture energy. For the UHPC containing the PPF, the fracture energies of 1.08–2.64 were attained at PPF content of 0.5%–2% at room temperature. The fracture energy of the UHPC reinforced with SF increases more quickly than that of the UHPC with PPF due to the fact they are efficiently anchored. As the temperature increased, the crack energy dropped significantly, but larger decreases were recorded for the UHPC reinforced with PPF.

Bencardino et al. [57] investigated the effect of 1% and 2% PPF and SF content on the fracture energy of high-performance concrete. The fracture energy equals 1.1 and 1.2 N/mm, at 1% and 2% of PPF content, as well as 5.2 N/mm at 1% of SF and 6.8 N/mm at 2% of SF. Comparing the above values of fracture energy with the results at room temperature given in Table 5, it can be concluded that in this study, higher values were obtained due to the higher strength of the concrete matrix and the smaller size of the specimens.

### 3.4. Strains and Macro-Crack Development

**Figure 11:**
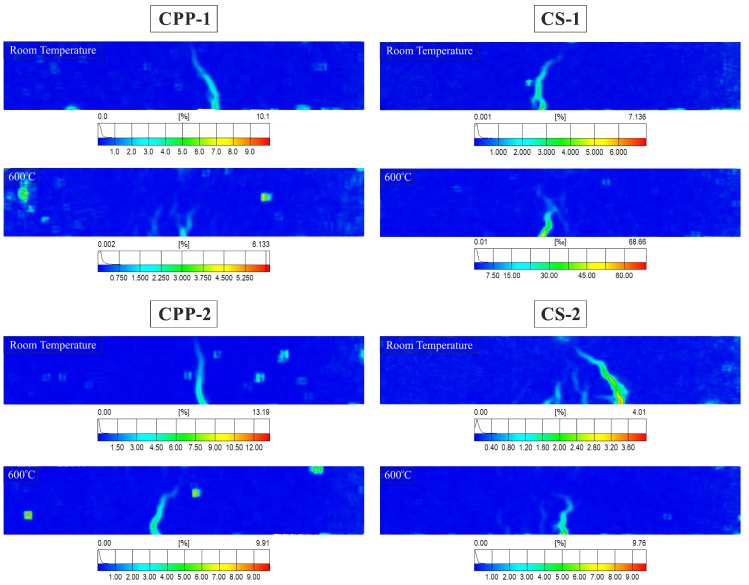
Von Mises strains in PPF- and SF-reinforced UHPC specimens at peak load with 1% and 2% fibre content at room temperature and after exposure to 600 °C.

The tensile fracture of fibre-reinforced concrete can be separated into the elastic stage, micro-cracking, stages of macro-crack growth, and the bridging stage of macro-crack [58,59]. In the first stage, all the specimens have linear elastic behaviour. During the micro-cracking stage in fibre-reinforced concrete, micro-cracks will be formed (detailed in Section 3.5). When loading of the specimens continues, the micro-cracks are propagated, connected, and form bigger macro-cracks, which have a length several times greater than the maximum grain size of aggregate. The macro-crack bridging stage starts when the deformation is concentrated in a narrow zone, with a single main crack. Figure 11 shows the location of the crack at peak load in PPF- and SF-reinforced UHPC with a 1% and 2% fibre content at room temperature and after exposure to 600 °C by means of plotting the von Mises strains in the three-point bending specimens measured by the Digital Image Correlation DIC) system (ARAMIS v6, GOM mbH, Braunschweig, Germany). The strain contours should be interpreted sensibly. The von Mises strain results are calculated from the average displacements. It means that the displacements are responsible for the high values of strains in the crack location zone. In fibre-reinforced concrete, the strain in a crack can be used not only to identify crack patterns, but also to determine the total crack bridging strain.

In these analyses of the efficacy of the fibres in controlling cracking and crack propagation at flexural load after exposure to elevated temperature, the non-contact ARAMIS-DIC system for 3D-measurements of deformation was used.

In the case of the PPF-reinforced UHPC specimen at room temperature, there was a sudden fall in the transfer of the tensile stresses from the concrete matrix to the PPFs during single macro-crack propagation within the mid-span. This is a consequence of the rather weak interface zone between the UHPC matrix and the PPF, which causes an inefficient transfer of stress from the UHPC to PPF. On the other hand, the UHPC with PPFs after exposure to a temperature of 600 °C transferred tensile stress by up to 90% less tensile stress compared to that achieved at room temperature. In this case, the single macro-crack split into several smaller cracks, connecting the tunnels formed after melting most of the PPFs. After exposure to elevated temperature, random surface defects are visible on the strain contours of the specimen, which indicates local cavities of the material during its heating.

In the SF-reinforced UHPC specimen at room temperature, the hooked-end SF–matrix bond was much better, but the number of SFs was lower than in the UHPC with PPF. The tensile behaviour after the first macro-crack formation remained steady and the tensile stress increased after the first cracking occurred. In each examined specimen, the first macro-crack was not the dominant crack. At a small distance from the first crack, another macro-crack was formed, which propagated until the specimen was damaged and partially closed the first macro-crack. CS-2 failed by creating fine multiple cracking in the bottom layer of the specimen. The SF-reinforced UHPC, unlike the PPF-reinforced UHPC, did not lose much of its tensile carrying capacity at 600 °C. Since the steel fibres did not melt or evaporate at this temperature, all of them remained intact and helped in carrying the stress. Multiple cracking was also observed in this case, which is the consequence of an improved fibre–matrix bond and the quite large number of fibres in the fracture regions.

**Figure 12:**
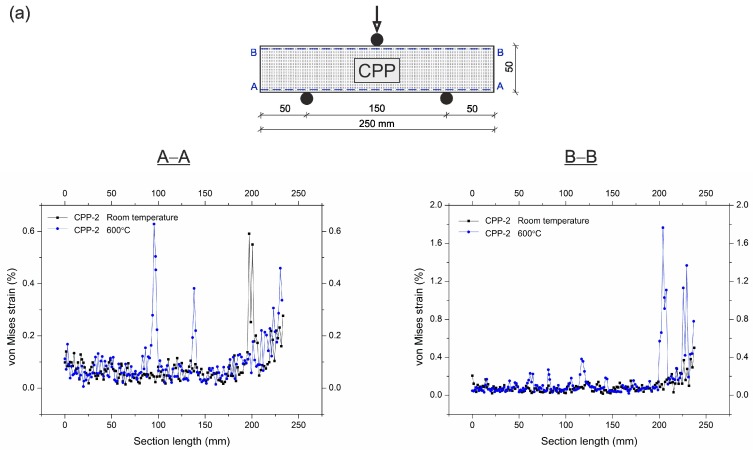

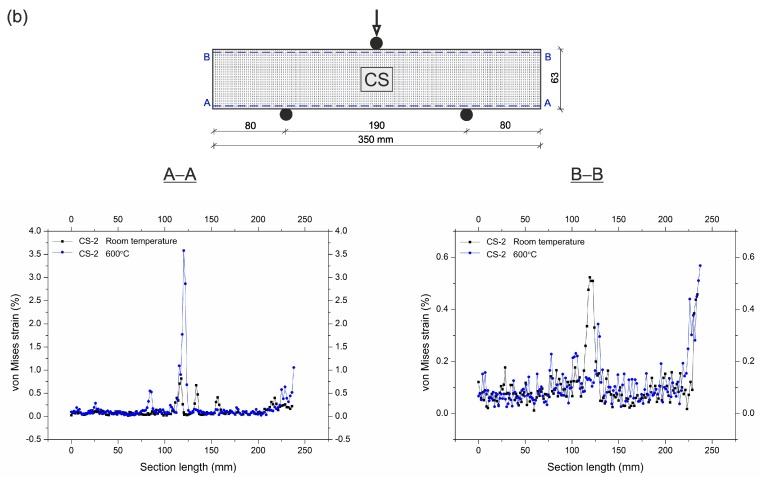
Comparison of von Mises strains at peak load, and at room temperature as well as after exposure to 600 °C in UHPC specimens with 2% (**a**) polypropylene fibre or (**b**) steel fibre content.

The von Mises strain curves from the beam specimens with the location of longitudinal cross-sections A–A and B–B are shown in Figure 12. The total crack bridging strains in the CPP-2 specimens at peak load are 0.64% at room temperature and 0.6% after exposure to 600 °C in the tension zone. On the other hand, these strains in the CS-2 specimens at peak load at the corresponding temperatures and tension zone are 3.7% and 0.8%, respectively. By contrast, in the compression zone, the total strains confining crushing in the CPP-2 and CS-2 specimens are 0.4% and 0.5% at room temperature, and are 1.8% and 0.6% after exposure to 600 °C, respectively. It can be noted that the most beneficial deformation distribution at peak stress was for the CS-2 specimen, in which the steel fibres bridged the macro-tensile cracks and confined the crushing zones, and provided a restricting effect up to failure in the tension and compression areas. The higher strain values at 600 °C indicated that the fibres had taken over the loading from the beginning due to the low strength of the UHPC matrix after its exposure to the elevated temperature. The SF increased the possibility of redistributing the stresses in the cracking and crushing regions, and improved the ductility of the specimens.

### 3.5. Microstructure

**Figure 13:**
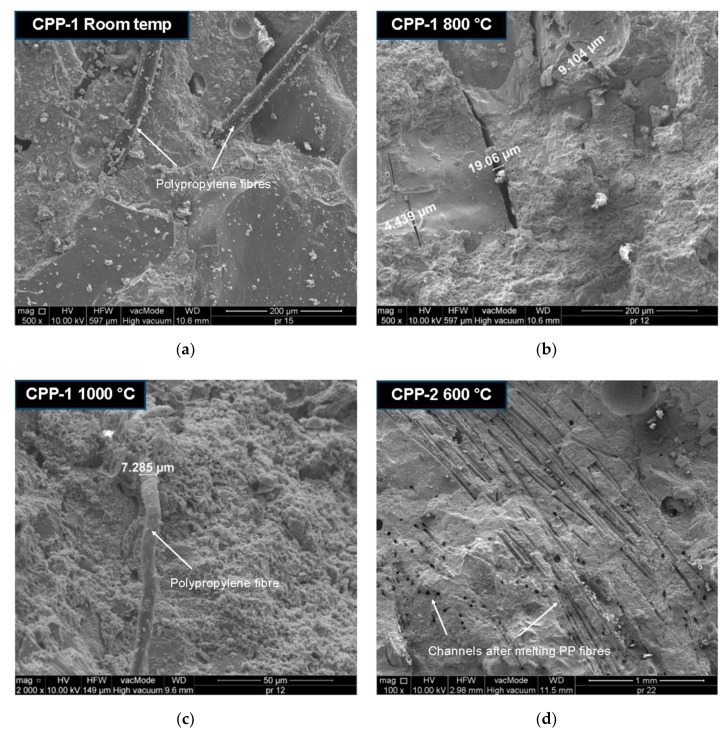

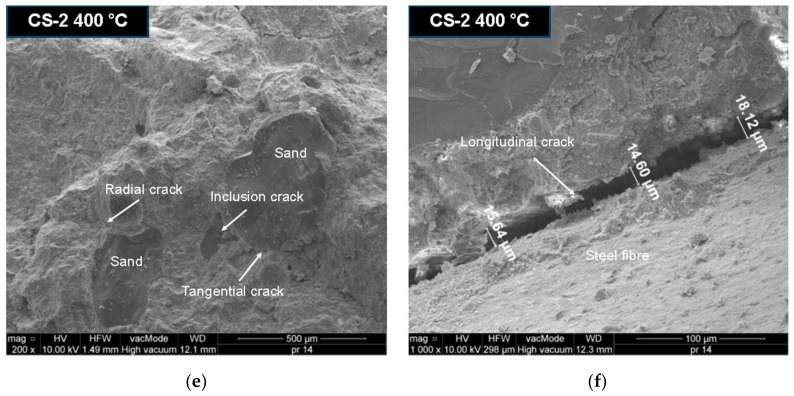
SEM micrographs of fibre-reinforced UHPC specimens after exposure to different high temperatures. (**a**) Morphology of specimen at room temperature, (**b**) extensive micro-cracks after exposure to 800 °C, (**c**) partially molten PPF after exposure to 1000 °C, (**d**) micro-channels after PPF melting, (**e**) typical micro-cracks after exposure to 400 °C, and (**f**) micro-crack at steel fibre–cement paste interface.

The thermally-damaged and control specimens were observed by scanning electron microscopy (SEM) at different magnifications to find evidence of a different resisting mechanism in the SF- and PPF-reinforced UHPC after high temperature exposure. Figure 13 presents SEM micrographs of the SF- and PPF-reinforced UHPC specimens with different contents of fibres after exposure to elevated temperatures. There are obvious micro-cracks on the failure surfaces of these composites. There are marked differences in the damage surfaces of the different fibre-reinforced UHPCs. The efficient control of micro-cracks development in the case of PPF-reinforced UHPC with 1% fibre content at room temperature is shown in Figure 13a. The UHPC specimen at room temperature had a good framework of plate calcium hydroxide crystals as needle or fibre C-S-H, ettringite crystals, and small pores. The fibres are randomly spaced in small increments, and their diameter is in the range of 14–40 µm. In general, the smaller the spacing between the fibers, the slower the micro-crack propagation will be, and as a result, a composite with a higher tensile strength should be obtained [58]. When UHPC is subjected to elevated temperatures, the incompatibility of thermal deformation of the UHPC constituents causes micro-cracks at the interfaces of the cement paste/aggregate and cement paste/fibre to open and propagate. In addition, internal stress caused by the build-up of steam pressure inside the pores results in changing the UHPC crystal structure as well as its volume. The dehydration of calcium hydroxide also leads to the formation of shrinkage cracks. Figure 13b shows the extensive micro-crack pattern of PPF-reinforced UHPC specimens after exposure to the temperature of 800 °C. The SEM micrographs show that the micro-cracks at the aggregate–cement paste interface are wider after exposure to 800 °C than at room temperature. In a severely damaged specimen after exposure to a high temperature, cracks run through the cement paste connecting the pores or go around it. Sometimes, cracks occur in the aggregate, with a higher concentration at the edge of the grains. Partially molten polypropylene fibres were even found in the CPP-1 specimen heated to 1000 °C, as shown in Figure 13c. In this case, the diameter of the fibre decreased by about 50%. The polypropylene fibres started to melt above 170 °C. Most of the PPFs had completely melted at 600 °C, leaving a network of micro-channels, as illustrated in Figure 13d. It can be seen from Figure 13e that three typical micro-cracks (radial cracks, tangential cracks, and inclusion cracks) around the sand aggregates particles appear in SF-reinforced UHPC, as one would expect. The longitudinal micro-crack at the interface of the steel fibre–cement paste in the SF-reinforced UHPC specimen (CS-2) after exposure to temperature of 400 °C is depicted in Figure 13f. It has been reported [60,61,62] that radial cracks in the cement paste will occur when the thermal expansion coefficient of the paste is smaller than that of the aggregate. By contrast, inclusion cracks in the aggregate and tangential cracks at the cement paste–aggregate interface will occur when the thermal expansion coefficient of the paste is greater than that of the aggregate. Li et al. [63] also reported that micro-cracks appeared at the interface between the paste and aggregate after high-temperature exposure. It was found [64] that sand usually had a lower linear thermal expansion coefficient at temperatures below 100 °C than cement paste. Therefore, tangential and inclusion cracks ought to appear at a temperature less than 100 °C, and radial cracks should be created at higher temperatures from the range of 200–300 °C. These cracks were developed from the thermal incompatibility between the sand aggregate and cement paste. The thermal decomposition of the hydration products and good dehydration-induced micro-cracks were also present in the UHPC matrix. Consequently, thermally induced damage of the mortar specimens is expected to be more severe than that of the paste specimens at elevated temperatures. When comparing the micro-cracks widths in UHPC reinforced PPF (CPP-1) and SF (CS-2), it can be noticed that even at a temperature lower than 400 °C in the steel fibres-reinforced concrete, micro-cracks of a similar width were formed, as in the concrete reinforced with polypropylene fibres. In this study, the micro-crack widths were between 4 and 20 µm. The experimentally observed micro-cracks widths in the fibre-reinforced concrete varied from 1.5 to 13.6 µm at room temperature [65,66].

## 4. Conclusions

The following conclusions based on the results and discussion presented in this study are given below.

1. The response of fibre-reinforced UHPC is dependent on the response of the UHPC matrix before the first peak load.
2. The experimental results showed that in some cases, the load–deflection curve of fibre-reinforced UHPC is a double-peak response. The first peak represents the properties of the UHPC matrix while the second peak represents the properties of the fibres. Under flexural load, the toughness decreases as the temperature increases.
3. The type of fibres and their content play an important role. After exposure to elevated temperatures, the hooked-end steel fibres changed colour, but they are still able to bridge the cracks and transfer the stress across the cracks. On the other hand, the polypropylene fibres evaporate and create channels for pressure to escape from UHPC subjected to elevated temperatures, so as to prevent its cracking and spalling. However, large drops in the load–deflection responses and toughness are observed for polypropylene fibre-reinforced UHPC when the temperature approaches 600 °C.
4. The toughness indexes of the SF-reinforced UHPC decrease nonlinearly with increasing temperature at a fibre content below 1%. By contrast, the toughness indexes of the PPF-reinforced UHPC decreases nonlinearly with increasing temperature up to about 400–500 °C, and then increases nonlinearly up to a temperature of 800 °C regardless of the fibre content.
5. The fracture energy decreases with the increasing temperature, but larger drops are obtained by UHPC reinforced with polypropylene fibres.
6. Steel fibres increase the strains in the UHPC as well as improve the ductility after exposure to elevated temperatures to a greater degree than polypropylene fibres.
7. The SEM observation results showed that the thermal damages of fibre-reinforced UHPC depends on the pore pressure effect, the thermal incompatibility between the constituents, the decomposition of hydration products, and the formation of micro-cracks.

## Figures and Tables

**Table 1:** Physical and chemical properties of the cement and condensed silica fume.

Material Characteristics	CEM I 52.5 N-HSR/NA	Condensed Silica Fume
Specific surface area (m^2^/kg)	443	17,000
Water demand (%)	30	–
Start of setting (min)	120	–
End of setting (min)	180	–
Volume stability acc. to Le Chateliere (mm)	2	–
Compressive strength at 2 days (N/mm^2^)	27.7	–
Compressive strength at 28 days (N/mm^2^)	57.1	–
Tensile strength at 2 days (N/mm^2^)	5.3	–
Tensile strength at 28 days (N/mm^2^)	8.2	–
Composition (%)	–	–
SiO_2_	20.92	94.80
Al_2_O_3_	3.50	1.30
Fe_2_O_3_	4.38	0.83
CaO	64.69	0.56
MgO	1.20	0.71
SO_3_	3.07	–
K_2_O	0.38	1.26
Na_2_O	0.22	0.41
Cl	0.08	–
Loss on ignition	1.27	0.12
Insoluble matter	0.20	–

**Table 2:** Grading of fine and coarse aggregates.

Sieve Size	Percentage Passing	
(mm)	Fine Aggregate	Coarse Aggregate
16	–	100.0
8	–	67.0
4	–	26.3
2	100	4.1
1	88.2	–
0.5	74.8	–
0.25	11.6	–
0.125	–	–
< 0.125	–	–

**Table 3:** Ultra-high performance concrete (UHPC) mixture proportion and properties of fresh concrete.

Designation	C0	CPP-0.5	CPP-1	CPP-1.5	CPP-2	CS-0.5	CS-1	CS-1.5	CS-2
Fibre volume content (%)	–	0.5	1.0	1.5	2.0	0.5	1.0	1.5	2.0
Cement (kg/m^3^)	670.5	670.5	670.5	670.5	670.5	670.5	670.5	670.5	670.5
Silica fume (kg/m^3^)	74.5	74.5	74.5	74.5	74.5	74.5	74.5	74.5	74.5
Fine aggregate (kg/m^3^)	500	500	500	500	500	500	500	500	500
Coarse aggregate (kg/m^3^)	990	990	990	990	990	990	990	990	990
Water (l/m^3^)	178	178	178	178	178	178	178	178	178
Superplasticiser (l/m^3^)	20	20	20	20	20	20	20	20	20
Steel fibre (kg/m^3^)	–	–	–	–	–	39	78	117	156
Polypropylene fibre (kg/m^3^)	–	4.5	9	13.5	18	–	–	–	–
Slump (mm)	120	102	91	83	72	109	99	91	84
Unite weight (kg/m^3^)	2434	2441	2447	2453	2458	2475	2514	2554	2594

Note: C0 = plain concrete; Ca-b = fibre-reinforced concrete, where a = fibre type, and b = fibre volume content (in %).

**Table 4:** Properties of hardened UHPC.

Designation	C0	CPP-0.5	CPP-1	CPP-1.5	CPP-2	CS-0.5	CS-1	CS-1.5	CS-2
Apparent density (g/cm^3^)	2.56	2.41	2.37	2.30	2.27	2.50	2.54	2.58	2.60
Absorbability (%)	0.6	0.9	0.9	0.8	0.7	1.8	2.0	2.1	2.5
Open porosity (%)	4.3	4.7	4.0	3.9	3.8	4.6	5.1	5.5	5.7
Compressive strength (N/mm^2^)	139.8	131.4	118.5	115.8	114.7	134.1	135.1	138.7	139.6
Splitting tensile strength (N/mm^2^)	9.4	10.5	10.2	9.9	9.4	10.6	11.7	13.1	11.8
Flexural strength (N/mm^2^)	7.7	8.0	7.0	6.5	5.6	8.3	9.4	11.1	9.6
Modulus of elasticity (N/mm^2^ × 10^3^)	48.6	43.7	42.6	40.9	40.0	50.2	50.7	51.1	51.6

**Table 5:** Peak loads, flexural toughness parameters, and fracture energy.

Designation	Temperature	Peak Load (kN)		Area Under the Curve (kN×mm) Up to				Toughness Index			Fracture Energy
	(° C)	1^st^ Peak	2^nd^ Peak	*δ*	3*δ*	5.5*δ*	10.5*δ*	I5	I10	I20	(N/mm)
CPP-0.5	Room	5.6	–	1.2	2.2	2.5	2.7	1.8	2.1	2.3	1.08
	400	3.5	–	0.8	0.9	1.1	–	1.1	1.4	–	0.44
	600	2.7	–	0.6	0.7	0.8	–	1.2	1.3	–	0.32
	800	1.1	–	0.3	0.4	0.6	–	1.3	2.0	–	0.24
CPP-1	Room	5.8	–	1.0	2.5	3.2	3.6	2.5	3.2	3.6	1.44
	400	3.2	–	0.7	0.7	0.8	–	1.0	1.1	–	0.32
	600	2.5	–	0.4	0.7	0.8	–	1.7	2.0	–	0.32
	800	0.7	–	0.2	0.6	0.7	–	3.0	3.5	–	0.28
CPP-1.5	Room	6.1	3.2	0.7	2.8	4.3	4.9	4.0	6.1	7.0	1.96
	400	3.0	–	0.3	0.8	0.9	–	2.7	3.0	–	0.36
	600	2.2	–	0.2	0.7	0.8	–	3.5	4.0	–	0.32
	800	0.6	–	0.1	0.5	0.6	–	5.0	6.0	–	0.24
CPP-2	Room	5.8	3.1	0.8	3.2	5.4	6.6	4.0	6.8	8.3	2.64
	400	2.6	–	0.4	0.8	0.9	–	2.0	2.3	–	0.36
	600	2.0	–	0.3	0.8	0.9	–	2.7	3.0	–	0.36
	800	0.6	–	0.2	0.6	0.7	–	3.0	3.5	–	0.28
CS-0.5	Room	5.8	7.5	0.7	6.1	11.5	16.8	8.7	16.4	24.0	5.33
	400	2.9	–	0.5	3.3	6.2	9.9	6.6	12.4	19.8	3.14
	600	2.2	–	0.4	1.9	3.1	5.8	4.8	7.8	14.5	1.84
CS-1	Room	6.8	8.7	1.0	7.9	14.7	22.7	7.9	14.7	22.7	7.21
	400	4.0	–	0.8	4.1	9.5	14.8	5.1	11.9	18.5	4.70
	600	3.3	–	0.6	2.5	4.6	8.4	4.2	7.7	14.0	2.67
CS-1.5	Room	12.8	11.7	4.3	17.8	31.2	42.6	4.1	7.3	9.9	13.52
	400	9.2	–	2.4	10.9	21.8	29.3	4.5	9.1	12.2	9.30
	600	8.3	–	1.6	8.0	15.5	21.9	5.0	9.7	13.7	6.95
CS-2	Room	11	10.9	3.6	17.1	30.3	49.2	4.8	8.4	13.7	15.62
	400	8.1	–	1.9	10.3	18.9	31.2	5.4	9.9	16.4	9.90
	600	7.4	–	1.0	7.2	12.6	19.3	7.2	12.6	19.3	6.13

**Table 6:** Temperature–toughness index relationship of SF/PPF-reinforced UHPC.

Designation	Equation	Residual Sum of Squares	Adj. R-Square
CPP-0.5	$I5=2.707\times 10^{-6}T^{2}-0.003T+1.846$	0.008	0.914
	$I10=4.848\times 10^{-6}T^{2}-0.004T+2.199$	0.030	0.820
CPP-1	$I5=1.108\times 10^{-5}T^{2}-0.008T+2.651$	0.014	0.981
	$I10=1.406\times 10^{-5}T^{2}-0.011T+3.392$	0.049	0.960
CPP-1.5	$I5=1.146\times 10^{-5}T^{2}-0.008T+4.148$	0.008	0.991
	$I10=1.933\times 10^{-5}T^{2}-0.016T+6.386$	0.053	0.977
CPP-2	$I5=8.479\times 10^{-6}T^{2}-0.008T+4.110$	0.206	0.701
	$I10=1.691\times 10^{-5}T^{2}-0.018T+7.082$	0.426	0.893
CS-0.5	$I5=-5.989\times 10^{-6}T^{2}-0.003T+8.763$	―	―
	$I10=-2.151\times 10^{-5}T^{2}-0.001T+16.438$	―	―
	$I20=-2.663\times 10^{-5}T^{2}+1.334T+24.008$	―	―
CS-1	$I5=4.946\times 10^{-6}T^{2}-0.009T+8.087$	―	―
	$I10=-2.350\times 10^{-5}T^{2}-0.003T+14.659$	―	―
	$I20=-1.974\times 10^{-5}T^{2}-0.003T+22.763$	―	―
CS-1.5	$I5=2.495\times 10^{-6}T^{2}+4.537\times 10^{-6}T+4.099$	―	―
	$I10=-2.995\times 10^{-6}T^{2}-0.006T+7.181$	―	―
	$I20=2.495\times 10^{-6}T^{2}+0.005T+9.799$	―	―
CS-2	$I5=1.279\times 10^{-5}T^{2}-0.004T+4.871$	―	―
	$I10=1.647\times 10^{-5}T^{2}-0.003T+8.453$	―	―
	$I20=1.275\times 10^{-5}T^{2}+0.002T+13.660$	―	―
